# Sequencing of an Undifferentiated Carcinoma with Osteoclast-Like Giant Cells of the Pancreas: A Case Report

**DOI:** 10.1089/pancan.2021.0004

**Published:** 2021-10-07

**Authors:** Jessica L. Smith, Christina L. Jacovides, Catherine M. Tucker, Wei Jiang, Anthony J. Prestipino, Charles J. Yeo

**Affiliations:** ^1^ Department of Surgery, Thomas Jefferson University Hospital, Philadelphia, Pennsylvania, USA.; ^2^ Division of Trauma, Surgical Critical Care and Emergency Surgery, Penn Presbyterian Medical Center, Philadelphia, Pennsylvania, USA.; ^3^ Department of Pathology, Anatomy, and Cell Biology, Thomas Jefferson University Hospital, Philadelphia, Pennsylvania, USA.

**Keywords:** pancreatic cancer, cancer, pancreatic adenocarcinoma, osteoclast-like giant cells, next-generation sequencing

## Abstract

**Background:** Undifferentiated carcinoma with osteoclast-like giant cells/osteoclast-like giant cell reaction (UC-OGC) is a rare form of pancreatic cancer historically associated with a poor prognosis. Molecular tumor profiling provides new information about tumor origins and a more nuanced understanding of the potential efficacy of different chemotherapeutic agents.

**Presentation:** A 69-year-old man presented with a 13-cm periampullary pancreatic mass. Biopsy of a neighboring lymph node was consistent with adenocarcinoma. After neoadjuvant chemoradiation, the patient underwent resection and the tumor was consistent with UC-OGC. Next-generation sequencing was performed with genomic and proteomic analyses analyzed by a molecular tumor board review. These analyses revealed genetic alterations similar to those seen in pancreatic ductal adenocarcinoma, as well as potential therapeutic targets for the patient's subsequent therapy.

**Conclusions:** Understanding a tumor's genetic changes allows for better understanding of its biology and may improve treatment efficacy. We believe that future study in tumor profiling will improve our understanding of rare cancers such as UC-OGC and also pave the way for the use of novel therapies to specifically target mutations in a broad range of more common tumors.

## Case Report

A 69-year-old male presented with abdominal distention, steatorrhea, and jaundice. Workup revealed a 13-cm periampullary pancreatic mass with an associated 13-mm cystic nodule. Biopsy of a neighboring lymph node demonstrated adenocarcinoma. The patient underwent biliary stenting and neoadjuvant chemoradiation with modified FOLIFIRINOX. Restaging imaging did not show liver metastases. He underwent pylorus-preserving pancreaticoduodenectomy with resection of a 6.6-cm exophytic pancreatic mass.

**FIG. 1.**
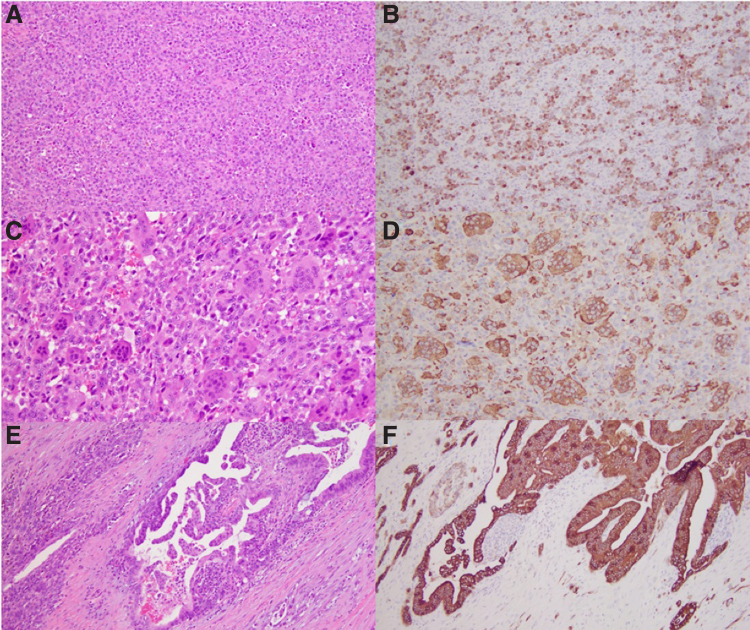
**(A, B)** Undifferentiated carcinoma (**A**, hematoxylin and eosin stain, 10 × objective) with diffuse involvement by pleomorphic mononuclear cells, which were positive for cytokeratins AE1/AE3 (**B**, 10 × objective). **(C, D)** Malignant mononuclear epithelial cells (**C**, hematoxylin and eosin stain, 20 × objective) were accompanied by numerous non-neoplastic multinucleated histiocytic cells that were positive for CD68 (**D**, 20 × objective). **(E, F)** Well-differentiated ductal adenocarcinoma component (**E**, hematoxylin and eosin stain, 10 × objective) highlighted by cytokeratin AE1/AE3 immunohistochemical stain (**F**, 10 × objective).

Histopathological evaluation of the tumor (Fig. 1) revealed a mostly undifferentiated pancreatic carcinoma composed of malignant pleomorphic mononuclear epithelial cells (A) accompanied by numerous non-neoplastic multinucleated giant cells (C). A well-differentiated pancreatic ductal adenocarcinoma (PDAC) comprised a minor component (E, F). The tumor was consistent with undifferentiated carcinoma with osteoclast-like giant cells/osteoclast-like giant cell reaction (UC-OGC), grade III (according to the Netherlands Committee on Bone Tumours grading system).^1^ On immunohistochemical staining, undifferentiated mononuclear carcinoma cells were positive for cytokeratin AE1/AE3 and CK7 (B). Multinucleated giant cells were negative for cytokeratins but positive for the histiocytic marker CD68 (D).

In-house solid tumor-focused gene mutational panel using next-generation sequencing was performed on tumor microdissected from formalin-fixed paraffin-embedded tissue block. This panel provides comprehensive detection of somatic mutations, including single nucleotide variants and small insertions/deletions up to 30 base pairs, in 30 important cancer-related genes. *KRAS* G12D and *TP53* C176F pathogenic mutations as well as an *IDH2* G144A mutation of unknown significance were identified.

Molecular profiling (genomic and proteomic analyses) performed by Perthera, Inc. (McLean, VA) evaluated the tumor for 315 cancer-related genes and introns from 28 genes frequently altered in cancer. The tumor's specific amplification of *KRAS* and presence of the activating mutation G12D as well as PD-L1 expression indicated that immunotherapy with an MEK/ERK (drug that inhibits the MEK/ERK proteins along the dysregulated Mitogen-activated protein kinase [MAPK] pathway) inhibitor would potentially be effective. Profiling suggested possible decreased sensitivity to standard PDAC combinations (e.g., gemcitabine plus nab-paclitaxel [TS positive and ERCC1 high] and 5-FU–based therapies [TS positive]).

Unfortunately, the patient developed biopsy-proven UC-OGC liver metastases 3 months postoperatively, consistent with the known poorer prognosis with a grade III/IV primary tumor.^1^

## Discussion

UC-OGC represents <1.4% of all pancreatic cancers.^1^ Earlier studies that did not distinguish between undifferentiated carcinoma and UC-OGC suggested that UC-OGC portends a significantly worse prognosis than PDAC.^2^ More recent comparisons suggest a more favorable prognosis,^1,3^ although higher grade tumors are associated with poorer outcomes.^1^

Histologically, UC-OGC is characterized by the presence of osteoclastic giant cells (OGCs) as well as both neoplastic and non-neoplastic mononuclear cells,^4^ all of which often coexist with invasive adenocarcinoma.^1^ Immunohistochemical staining suggests that these infiltrating neoplastic mononuclear cells arise from duct epithelium^5^ and are the true malignant culprit in UC-OGC, whereas the OGCs represent reactive non-neoplastic cells recruited to the tumor.^1,4^ Similar tumorigenesis pathways have been identified in other solid organ sarcomas.^1^ Achieving a better understanding of the genes and proteins involved in these pathways may improve our understanding of the pathogenesis of these tumors.

Pancreatic cancer patients who undergo molecular tumor profiling and receive targeted therapies survive longer than those who do not receive targeted therapies.^6^ In this case, the patient's tumor profile suggested that it may be less sensitive to the two standard regimens for PDAC, but did suggest the tumor may be sensitive to novel regimens currently in clinical trials.

The rarity of UC-OGC and its unique biology involving the recruitment of osteoclast-like cells to the tumor bed make it challenging for prognosis, but intriguing from a cancer biology perspective. By harnessing the power of molecular profiling, we hope not only to refine our understanding and treatment of rare tumors such as UC-OGC, but also to highlight key molecular pathways for targeted cancer therapeutics.

## Abbreviations Used

FFPE: formalin-fixed paraffin-embedded tissue
OGC: osteoclastic giant cells
PDAC: pancreatic ductal adenocarcinoma
UC-OGC: undifferentiated carcinoma with osteoclast-like giant cells/osteoclast-like giant cell reaction
